# Lead Management Decision-making: What Can We Learn from Outcomes Following Lead Extraction versus Capping?

**DOI:** 10.19102/icrm.2018.090306

**Published:** 2018-03-15

**Authors:** Jonathan P. Piccini

**Affiliations:** ^1^Duke University Medical Center, Durham, NC, USA; ^2^Duke Clinical Research Institute, Durham, NC, USA

**Keywords:** Implantable cardioverter-defibrillator, lead extraction, lead management, pacemaker

## Introduction

Lead management is an increasingly important aspect of care in patients with cardiac implantable electronic devices; however, relatively little is known about the long-term outcomes after capping and abandoning leads.

An analysis study, *Outcomes Associated with Extraction versus Capping and Abandoning Pacing and Defibrillator Leads*, was designed to compare lead capping and abandonment versus extraction in Medicare patients without device infections and with pacemakers or implantable cardioverter-defibrillators undergoing lead addition. It had been hypothesized that a cap-and-abandon strategy would be associated with lower short-term mortality, while lead extraction would be associated with better long-term outcomes.

We speak with Dr. Jonathan Piccini, the senior author of the study,^1^ who provides his perspectives surrounding the major findings and clinical implications of the published results.

## About Dr. Piccini

Jonathan P. Piccini, MD, MHS, FHRS, is a clinical cardiac electrophysiologist and Associate Professor of Medicine at Duke University Medical Center and the Duke Clinical Research Institute. His research interests include the conduction of clinical trials and the assessment of cardiovascular therapeutics for the care of patients with heart rhythm disorders. At present, he is the Director of the Duke Center for Atrial Fibrillation. Dr. Piccini has more than 250 publications in the field of heart rhythm medicine. Clinically, his focus is on the care of patients with atrial fibrillation and complex arrhythmias, with particular emphasis on catheter ablation and lead extraction.

## Interview

***Question:*** Thank you for taking the time to participate in this discussion, Dr. Piccini. For the first question, could you answer how many physicians participated in this study?

***Piccini:*** This study was not a prospective study and so there were no enrolling physicians. We performed a retrospective cohort study using nationwide Medicare data from the United States. Our clinical research team involved a large number of biostatisticians and clinical investigators. The clinical investigators included lead extraction specialists and electrophysiologists from Duke University, the Duke Clinical Research Institute, Brigham and Women’s Hospital, and the University of Miami.

***Question:*** How many patients were included in the study?

***Piccini:*** The analysis included 6,859 patients who underwent *de novo* cardiac implantable electronic device implantation between January 1, 2000 and December 31, 2013 who also underwent a subsequent lead addition or extraction ≥ 12 months after the implant. Among the 6,859 patients, 1,113 (16.2%) underwent extraction and 5,746 (83.8%) underwent capping and abandonment, respectively.

***Question:*** What was the length of the study?

***Piccini:*** One of the advantages of our study was the duration of the follow-up. Many prior studies of lead management have focused on 30-day or one-year outcomes. We were able to look at outcomes beyond one year. The median follow-up was 2.4 years, but we were able to report follow-up outcomes out to five years in many patients.

***Question:*** Who funded the study?

***Piccini:*** The analysis was funded by a grant from Spectranetics given to the Duke Clinical Research Institute.

***Question:*** What were this study’s major findings?

***Piccini:*** There were three main findings. First, we found that rates of device infections across the US were significantly higher than previous estimates from prior studies. Second, lead extraction was associated with lower adjusted five-year infection rates when compared with a cap-and-abandon strategy. Finally, we did not observe any evidence of increased mortality with lead extraction. In fact, absolute mortality was lower in those who underwent lead extraction, though there was no difference after adjustment for patient characteristics.

***Question:*** What would you say are this study’s clinical implications?

***Piccini:*** One of the challenges when counseling patients on their lead management options is that there are few data available regarding the long-term risks associated with either treatment strategy (extraction versus capping and abandoning). The results from our study show that there is no difference in short-term or long-term mortality with lead extraction versus with capping and abandoning. However, there was a lower long-term risk of infection with an extraction strategy. This information should be shared with patients undergoing shared decision-making.

***Question:*** Why should patients consider extraction versus capping or abandonment?

***Piccini:*** There are many advantages and disadvantages to each of these treatment options. One of the advantages to a cap-and-abandon strategy is that the risks of lead extraction are avoided. However, capping and abandoning hardware results in a higher hardware burden with increased risks of venous occlusion, long-term infection, and other lead-associated complications such as tricuspid regurgitation and lead-lead interactions. Extraction helps preserve the current implant vein by avoiding higher lead burden and an increased risk of venous occlusion, and also is associated with a lower risk of long-term infection. Additionally, abandoned leads are often considered as a contraindication to magnetic resonance imaging (MRI) imaging in most US centers. Many patients will require MRI imaging at some point in their lifetime. Again, the advantages and disadvantages always have to be considered in the context of each patient and his or her unique circumstances.

***Question:*** What factors do you personally consider in making a recommendation to a patient? Why?

***Piccini:*** Everything in lead management is patient-specific, so generalizations can pose difficulties. However, I favor lead extraction in younger patients and in patients in which a cap-and-implant strategy will require moving to the contralateral side (ie, venous occlusion).

***Question:*** How safe is lead extraction? What available evidence is there to support this? Is there direct evidence to support this?

***Piccini:*** There is often concern that lead extraction is associated with increased mortality. In cases of infection, lead extraction is actually a life-saving procedure. In elective cases, where capping is also an option, our data from US practice show no evidence of increased mortality with lead extraction. However, it is important to note that the periprocedural safety of lead extraction and outcomes are dependent upon the physician experience, back-up plans, and operator-specific outcomes at a given institution. For example, lead extraction performed by an inexperienced team without cardiothoracic surgical support is not a good situation for either the patient or the provider.

***Question:*** How does the safety of lead extraction compare with that of other cardiovascular procedures such as ablation? Is there any direct evidence to support this?

***Piccini:*** Perioperative risk and outcomes of lead extraction procedures are often over-stated and misunderstood. Nationwide data^2^ show that the risk of a major complication with lead extraction is less than that associated with percutaneous coronary intervention for acute myocardial infarction or catheter ablation of atrial fibrillation. Multicenter data from 13 centers in the LExICon study found that the periprocedural mortality for lead extraction procedures is 0.3%.^2^

***Question:*** What is Duke’s stance on capping versus abandonment?

***Piccini:*** We always consider lead management strategy on a patient-specific case-by-case basis. For example, shared decision-making for lead management is very different in a healthy 45-year-old versus an 83-year-old with multiple comorbidities. The risks of capping and the risks of extraction are considered for each specific patient. In general, in younger patients (those aged > 70 years), in whom the potential for long-term risks of capping are higher, we seriously consider the role of extraction. However, in older patients, in whom the potential for long-term risks of capping are limited, we seriously consider the use of a cap-and-abandon approach. However, it is crucial to emphasize that every patient is different and unique.

***Question:*** Can you describe Duke’s protocol for lead extraction (with respect to facility, work flow, team approach, and surgical backup)?

***Piccini:*** All of our lead extractions are performed in our hybrid operating room, which is in our cardiothoracic surgical suite. We have a cardiac surgeon immediately available in the room in the rare event that there is a need for a rescue sternotomy. The procedure is performed with the patient under general anesthesia and with the use of perioperative transesophageal echocardiography. We also type and cross four units of packed red cells, which are also immediately available in the room. With this back-up plan, we have a mortality rate of less than 0.1%.

***Question:*** Are there any additional thoughts that you would like to add?

***Piccini:*** I think it is also important to note that periprocedural imaging can contribute to improving perioperative risks and outcomes of lead extraction procedures. Several groups have shown^3–5^ that preprocedural computed tomography scanning and intracardiac echocardiography can identify high-risk anatomy that can help to facilitate procedural planning and extraction technique.
